# Anti-cancer effects of human placenta-derived amniotic epithelial stem cells loaded with paclitaxel on cancer cells

**DOI:** 10.1038/s41598-022-22562-w

**Published:** 2022-10-28

**Authors:** Amirhesam Babajani, Asma Manzari-Tavakoli, Elham Jamshidi, Roghayeh Tarasi, Hassan Niknejad

**Affiliations:** Department of Pharmacology, School of Medicine, Shahid Beheshti University of Medical Sciences, Tehran, Iran

**Keywords:** Biochemistry, Stem cells, Cancer, Cancer therapy

## Abstract

Available therapeutic strategies for cancers have developed side effects, resistance, and recurrence that cause lower survival rates. Utilizing targeted drug delivery techniques has opened up new hopes for increasing the efficacy of cancer treatment. The current study aimed to investigate the appropriate condition of primming human amniotic epithelial cells (hAECs) with paclitaxel as a dual therapeutic approach consisting of inherent anticancer features of hAECs and loaded paclitaxel. The effects of paclitaxel on the viability of hAECs were evaluated to find an appropriate loading period. The possible mechanism of hAECs paclitaxel resistance was assessed using verapamil. Afterward, the loading and releasing efficacy of primed hAECs were evaluated by HPLC. The anti-neoplastic effects and apoptosis as possible mechanism of conditioned media of paclitaxel-loaded hAECs were assessed on breast and cervical cancer cell lines. hAECs are highly resistant to cytotoxic effects of paclitaxel in 24 h. Evaluating the role of P-glycoproteins in hAECs resistance showed that they do not participate in hAECs resistance. The HPLC demonstrated that hAECs uptake/release paclitaxel with optimum efficacy in 8000 ng/ml treatment. Assessing the anti-proliferative effect of primed hAECs condition media on cancer cells showed that the secretome induced 3.3- and 4.8-times more potent effects on MCF-7 and HeLa, respectively, and enhanced the apoptosis process. These results suggest that hAECs could possibly be used as a drug delivery system for cancer treatment. Besides, inherent anticancer effects of hAECs were preserved during the modification process. Synergistic anticancer effects of paclitaxel and hAECs can be translated into clinical practice, which would be evaluated in the future studies.

## Introduction

Cancer is a heterogeneous disease with irregular cell division and the capability to invade and metastasize secondary sites from a primary tumor. Cancer imposes considerable mortality and disease burden on societies and health systems^1^. Based on the last evaluations, the world experienced about 19.3 million new cancer cases and nearly 10.0 million deaths due to cancer in 2020. The most frequently identified cancers were female breast cancer, with 2.26 million cases worldwide^2^. Many anticancer strategies have been administered to reduce cancer complications and mortality, such as radiotherapy, chemotherapy, hormone therapy, and surgery^3,4^.

As the first cancer therapeutic approach, chemotherapy is based on the systemic administration of cytotoxic drugs that affect the spread and progression of malignancies by interfering with intracellular biological pathways and impairing cell division. Various chemotherapeutic agents have been introduced to reduce the mortality and morbidity of several malignancies. In this regard, paclitaxel (PTX) is a microtubule-stabilizing chemotherapeutic agent which binds to N-terminal 31 amino acids of the β-tubulin subunit to immobilize microtubules and inhibit de-polymerization^5^. It has been shown that the concentration of PTX indicates whether it prevents mitosis (in low concentrations) or induces microtubule detachment from centrosome (in high concentrations)^6^. However, collateral toxicity, side effects, and multidrug resistance of chemotherapeutic drugs, especially PTX, have remained challenges in using this anti-neoplastic method^6–8^. Since most of these chemotherapeutic agents act untargeted, they affect normal cells and cause intolerable symptoms such as hair loss, bone marrow suppression, malabsorption, and pain, which reduce patient compliance. In addition, low accessibility to tumor sites and multidrug resistance progression have lessened the efficacy of standard chemotherapy^9^. A new insight into delivering drugs to cancerous cells has led to an emerging novel therapeutic approach in oncology, so-called "targeted therapy"^8^. Targeted drug delivery is appropriate for facilitating drug transport to the tumor and metastatic sites. In order to localize the effects of chemotherapeutic drugs in the tumor microenvironment (TME), many methodological approaches, from physical targeting through ultrasound and magnetic field to drug carrier systems such as antibody-drug conjugates (ADCs) and nanosystem drug carriers, have been studied^10,11^. One of the promising approaches for targeting cancer cells is utilizing cells as both therapeutic and drug carrier agents. Some studies have used mesenchymal stromal cells (MSCs) as a drug delivery platform to tumor sites. Although these cells possess some desired features, such as resistance against chemotherapeutic drugs, tumor targeting, immunomodulation, and apoptotic effects on cancer cells, utilizing MSCs for cancer treatment has faced undeniable challenges^12^. It has been demonstrated that MSCs encourage neoplastic cells toward a pro-metastatic and invasive state. They also persuade epithelial-mesenchymal transition (EMT) as a primary stage of cancer secondary metastasis^13^. Besides, MSCs have shown to enhance angiogenesis within the TME which assist tumor progression and metastasis^14,15^. To surmount the drawbacks of MSCs application in cancer treatment, it has been shown that epithelial cells located in the inner layer of the amnion membrane called human amniotic epithelial cells (hAECs) possess remarkable features that bring them up as appropriate candidates for cancer treatment.

The innate immune-privileged characteristics of hAECs provide a defensive barrier for the fetus to tolerate maternal immune system reactions. As the underlying reason, studies showed that hAECs express low amounts of human leukocyte antigens (HLA) such as HLA-A, HLA-B, HLA-C, and HLA-DR, whereas they express immunosuppressive HLA-G, which enhance their immune privilege ability. In this regard, the chance of immune rejection would be considerably reduced after transplantation of hAECs^16^. From the availability point of view, approximately 100 million hAECs could be isolated from each amnion membrane which would be expanded up to 10 to 60 billion cells after six passages^17^. Besides, isolation of similar cell numbers takes approximately 4–6 weeks for amniotic MSCs, while it takes about four hours for hAECs^16^. hAECs also possess innate anticancer effects. It has been shown that hAECs induce apoptosis in cervical cell carcinomas and triple-negative breast cancer (TNBC) cells through increasing caspase-3 and caspase-8 in tumor cells^18,19^. In addition, an in vivo study has shown that hAECs release transforming growth factor (TGF)-β1, which reduced the proliferation of epithelial ovarian cancer cells and caused G0/G1 cancer cell cycle arrest^20^. As angiogenesis plays a pivotal role in cancer supply and metastasis, hAECs also inhibit angiogenesis in TME through inhibition of heat shock protein-90 (HSP-90) and secretion of anti-angiogenic factors including thrombospondin, tissue inhibitors of matrix metalloproteinase (TIMP) -1, -2, -3, and -4, IL-10, collagen XVIII, endostatin, pigment epithelium-derived factor (PEDF) and IL-1 receptor antagonist^21^. As an essential feature, hAECs are not tumorigenic in mice who received these cells since they do not express telomerase as a critical factor in cell viability^17^. Besides, hAECs have entered clinical trials that shows their safety^22^. Considering these appropriate features, hAECs seem the proper candidate for suggesting a novel targeted anticancer delivery system. In the present study, we evaluated the effect of PTX, a microtubule stabilizer chemotherapeutic drug, on the viability of hAECs and the appropriate condition of PTX loading on hAECs. Besides, the PTX loading and releasing efficacy were assessed. Also, we investigated whether condition media of hAECs isolated from the human placenta after loading with paclitaxel (PTX) may display anti-proliferative effects on MCF-7 and HeLa cell lines.

## Methods

### Culture of cancer cell lines

Human cancer cell lines, MCF-7 and HeLa, were purchased from the Pasteur Institute. The cancer cell lines were cultured in RPMI (Sigma-Aldrich) media supplemented with 10% fetal bovine serum (FBS) (Sigma-Aldrich) and 100 U/ml penicillin/streptomycin (Thermo Fisher, USA). The incubation condition consists of 95% humidified air with 5% CO_2_ at 37 °C. The culture medium was replaced every three days, and cancer cells were passaged when they reached 70–80% confluency by 0.15% trypsin-EDTA (Merck, Germany).

### PTX sensitivity of hela and MCF-7 cell lines

The PTX sensitivity of Hela and MCF-7 cell lines was evaluated in 96-multiwell plates. In the beginning, 10,000 cells/well were seeded in 150 μl/well of culture medium (RPMI+FBS10%+100 U/ml penicillin/streptomycin) in an incubator with 95% humidified air with 5% CO_2_ at 37 °C. After reaching 80% confluence, the cells were then incubated for 24 hours with serial dilutions of PTX (Sobhan Oncology, Iran) for cytotoxicity test (500, 1000, 2000, 4000, 8000, 16000, and 32000 ng/ml). At the end of the incubation time, the cell viability was evaluated by the 3-(4,5-dimethylthiazol-2-yl)-2,5-diphenyltetrazolium bromide (MTT) assay.

### Isolation of hAECs from human amniotic membrane

hAECs were isolated based on the methods of our previous studies^23^. After Cesarean deliveries from healthy mothers and obtaining informed consent, the human placenta (n=16) was transferred to the laboratory under sterile conditions. All experimental procedures were done after approval by the ethics committee of Shahid Beheshti University of Medical Sciences (Approval ID: IR.SBMU.MSP.REC.1399.131). Besides, all methods were performed in accordance with the relevant guidelines and regulations. The amniotic layer was mechanically separated from the chorion layer and rinsed with cold phosphate-buffered saline (PBS) to remove blood and debris. Then, the amniotic membrane (AM) was dissected into small pieces and incubated with trypsin-EDTA enzyme 0.15% (Merck, Germany) at 37 °C for 10 minutes. Cells at this stage were discarded to remove debris. Supernatants were collected from the second and third 40 minutes of digestion, and trypsin was inactivated with FBS (Gibco, UK). The cell suspension was centrifuged at 1200 rpm for 12 minutes. Afterward, hAECs were suspended in Dulbecco Modified Eagle's Medium (DMED) (Gibco, UK) containing 100 U/ml penicillin/streptomycin (Thermo Fisher, USA) and 10% FBS (Sigma-Aldrich).

### Characterization of hAECs by immunocytochemistry

Immunocytochemistry was carried out to characterize the newly isolated hAECs at the protein expression level. We fixed the isolated hAECs at room temperature (25°–27 °C) in 4% paraformaldehyde for 10 min. Then, they were washed with PBS and incubated with 10% goat serum and 0.1% Triton X-100 for one hour. Prepared hAECs were treated with pan-cytokeratin primary antibody (Sigma, 1:200) overnight at 4 °C. We used a secondary antibody conjugated with rhodamine (Chemicon, 1:100) for 30 minutes after PBS washing. Staining nuclei with 4′,6-diamidino-2-phenylindole (DAPI) (Sigma, the USA) was used for counting the total number of cells.

### PTX sensitivity and proliferation of hAECs

The effects of PTX on the viability of hAECs (10^4^ cells) were evaluated in 96-multiwell plates by culturing the second passage of hAECs in 150 μl/well of culture medium (DMEM+FBS10%+100 U/ml penicillin/streptomycin) in the incubator with 95% humidified air with 5% CO2 at 37 °C. After reaching 80% confluency (about 48 hours), the cells were then incubated for 24, 48, or seven days with dilutions of PTX (500, 1000, 2000, 4000, 8000, 16000, 32000 ng/ml). For seven days of PTX treatment, the culture medium containing serial dilutions of PTX was replaced every 48 to maintain nutrient supplements. After a definite period, the plates were evaluated by MTT assay.

To evaluate the proliferative behavior of hAECs after paclitaxel priming, the proliferation of the second passage of hAECs after 24 hours of exposure to paclitaxel was assessed. Proliferation ability was evaluated in 96-multiwell plates by first seeding 2000 cells per well and treating the cells with dilutions of PTX (500, 1000, 2000, 4000, 8000, 16000, and 32000 ng/ml) for 24 hours. Afterward, the drug-containing medium was replaced with a fresh drug-free medium. hAECs were incubated for seven days, and the medium was replaced every two days. The viability was assessed by MTT assay.

### Mechanism of PTX resistance of hAECs

The effect of Verapamil (Hexal AG, Deutschland) as a *P*-glycoprotein (*P*-gp) efflux pump inhibitor was evaluated on the PTX resistance of hAECs through a proliferation assay as described above. hAECs were seeded in the presence of serial PTX concentrations and 20 μM (9092 ng/ml) of Verapamil^24^.

### PTX loading on hAECs

PTX loading was conducted by preculturing 10^5^ hAECs in a 6-well plate with 5 ml of complete medium for about 96 hours until the cells reached 80% confluence. Since the concentrations of 500 and 1000 ng/ml were insufficient for inducing toxicity in HeLa and MCF-7 cancer cell lines and also 32000 ng/ml of PTX induced considerable toxicity in hAECs after 24 hours which may impair the biological function of these cells, PTX was added to 5 ml of fresh medium (DMEM+FBS10%+100 U/ml penicillin/streptomycin) at final concentrations of 2000, 4000, 8000, 16000 ng/ml. The cells were incubated with PTX for 24 hours in an appropriate condition (37 °C + 5% CO_2_).

### PTX release by hAECs

PTX-loaded hAECs were detached from the 6-well plate with trypsinization (0. 2 % trypsin/EDTA), and the cells were washed twice in HBSS. After counting, PTX-loaded hAECs were seeded into a 6-well plate (untreated hAECs were cultured as the control group). After 24, 48, 72, and 96 hours of culture, the cell-conditioned medium (CM) was collected and replaced by a fresh medium. Collected CMs were centrifuged at 2500 rpm for 15 minutes to discard cell debris and stored at − 70 ℃^24^. The remained cells were trypsinized (0. 2% trypsin/EDTA) and then lysed through sonication by three cycles of 0.4 s pulse at 30% amplitude each (Probe sonicator, Hielscher Ultrasonics GmbH, Germany)^25^. After centrifugation of cell lysate at 2500 rpm for 15 minutes, cell debris was discarded, and the lysates (hAEC-PTX/LYS) were stored in PBS at− 70 ℃^24^.

### PTX measurement by high-performance liquid chromatography (HPLC)

A reversed-phase HPLC method was developed to determine the amount of paclitaxel in cell culture medium and cell lysate over the concentration range of 2000–16000 ng/mL. The samples were combined (1/4 v/v) with ethyl acetate, vortexed for 5 minutes, and centrifuged at 2500 rpm for 15 minutes. Then, an aliquot of 20 µL was injected into HPLC (MERCK-HITACHI). The HPLC system was equipped with an L7100 pump, two solvent inlets, a P5 ODS Hichrome (C18) column with a length of 250 mm, and an internal diameter of 4.6 mm filled with particles with dimensions of 5 microns and UV detector L-7420. Besides, data were collected using MULTI-HSM software. The chromatographic separation was performed with 1 ml/min flow rate. The mobile phase consisted of acetonitrile/water solution (65:35, v/v), and the detection wavelength was 230 nm. The concentration of PTX was calculated using a standard calibration curve experimentally. The paclitaxel retention time was 18 min. A calibration curve (200, 500, 1000, 2000, 4000, 8000, 16000 ng) in drug free medium was prepared and used to quantify paclitaxel (y = 38.843x–4607.3, R^2^ = 0.9999). We calculated the limit of quantification (LOQ) and limit of detection (LOD) by applying the formulae 1 and 2, respectively, which show that the standard deviation (σ) of the Y-intercepts is divided with slopes.

1 $$ LOD = \frac{{3.3 \times {\upsigma }}}{slope} $$

2 $$ LOQ = \frac{{10 \times {\upsigma }}}{slope} $$

Based on the standard curve, the concentrations of PTX in hAEC-PTX/CMs at priming concentrations of 2000, 4000, 8000, and 16000 ng/ml were calculated. Considering the number of cultured cells and the sample volume (ml), the amount of released PTX in hAEC-PTX/CMs (formula 3) and the amount of PTX that each hAEC released (ng) (formula 4) were calculated based on the following formulae.

3 $$ Released\, PTX \left( {ng} \right) = PTX\, released \,concentration \left( {\frac{ng}{{ml}}} \right) \times Sample\, volume\left( {ml} \right) $$

4 $$ PTX\, per \,cell \left( {pg} \right) = \frac{{Released\, PTX \left( {ng} \right) \times 10^{3} }}{{\text{Number\, of\, cultured\, cells}}} $$

Considering the initial volume of culture medium (5ml), hAECs were exposed to 2000 ng/ml (10000 ng), 4000 ng/ml (20000 ng), 8000 ng/ml (40000 ng), and 16000 ng/ml (80000 ng) of PTX, which is called PTX loading amounts (ng). Total drug uptake for each priming concentration was calculated by summing cumulative amounts of released PTX during 96 hours, and the remained PTX in hAEC-PTX/LYS (formula5). Also, total drug release (formula6) was assessed by adding cumulative amounts of released PTX during 96 hours. The release (formula7) and uptake (formula8) proportions were calculated based on the total drug release and total drug uptake ratios.

5 $$ Total\, PTX \,uptake \left( {ng} \right) = \sum PTX \,in \left( {hAECPTX - CMs} \right)\left( {ng} \right) + PTX \,in \left( {hAECsPTX - LYS} \right) \left( {ng} \right) $$

6 $$ Total \,PTX \,release \left( {ng} \right) = \sum PTX \,in \left( {hAECPTX - CMs} \right)\left( {ng} \right) $$

7 $$ PTX\, uptake \,propotion = \frac{{Total \,PTX \,uptake \left( {ng} \right)}}{{PTX \,loading \,amount\left( {ng} \right)}} $$

8 $$ PTX \,release \,propotion = \frac{{Total \,PTX \,release \left( {ng} \right)}}{{Total \,PTX \,uptake \left( {ng} \right)}} $$

### Anticancer effect of CM in vitro

The effect of pure PTX, hAECs-CM, and hAEC-PTX/CM on tumor cell proliferation was studied in 96-well plates utilizing HeLa and MCF-7 cell lines as target cells. The hAECs-CM and hAEC-PTX/CM were serially diluted with fresh culture medium in 1 to 2 ratios to the final volume of 160 μl of culture medium/well and added to 2000 tumor cells. After five days of culture at 37 °C, 5% CO_2_ cell proliferation was evaluated by the MTT assay. The anti-proliferative activity of hAEC-PTX/CM was compared to the pure PTX and hAECs-CM by comparing IV50 and IC50. IV50 is the volume of hAECs derived CM (μl/well) that 50% of inhibition was observed in which, and IC50 is the concentration (ng/ml) of pure PTX producing 50% of proliferation inhibition.

### Apoptosis analysis of cancer cells after exposure to CM of primed hAECs

HeLa and MCF-7 cancer cells (3 × 105 cells/well) were cultured in a completed medium (DMEM + FBS10% +  U/ml penicillin/streptomycin) in a 6-well multi-plate under 95% humidified air with 5% CO_2_ at 37 °C in incubator. After reaching 80% confluence, the DMEM culture medium was replaced with pure PTX (8000 ng/ml), hAECs-CM (1:1), and hAEC-PTX/CM (1:1) of 8000 ng/ml primed hAECs in separated experiment groups. Besides, the HeLa and MCF-7 cancer cells cultured in the completed medium (DMEM + FBS10% + 100 U/ml penicillin/streptomycin) were used as the control groups. For evaluation of cell apoptosis, after three days, the cells were collected and evaluated by Annexin-V and propidium iodide (PI) staining, utilizing the fluorescein isothiocyanate (FITC) Annexin V apoptosis detection kit (BD Biosciences, USA), based on the manufacturer's instructions. In this regard, cancer cells were detached by trypsin/EDTA 0.1%, washed twice with cold PBS, and centrifuged (2000 rpm, 5 min). We also added the culture medium on the treated cells in the centrifuge process to avoid missing detached and floating cells. After supernatant removal, the remaining cells were resuspended in 100 µl of binding buffer and incubated at room temperature (25°–27 °C) with Annexin-V FITC and PI solutions. Following the addition of 400 µl binding buffer, the cells were analyzed using a FACS Calibur Flow cytometer (BD Biosciences, the USA). The outcomes were reported as the percentage of Annexin V ^positive^ (early apoptosis), PI ^positive^ (necrosis), and Annexin V^positive^ PI ^positive^ (late apoptosis). Annexin V^negative^ PI^negative^ (double negative) was considered as viable cells.

### Statistical analysis

Graph Pad Prism software 8 was utilized for conducting the statistical analysis. Analysis of variance (ANOVA) test with Tukey's posttest was applied to compare the three experimental groups. Besides, data were represented as the mean ± standard deviation. A *P*-value less than 0.05 was considered to indicate a significant difference. Data of FACS were analyzed by CellQuest 3.3 (BD Biosciences, San Jose, CA).

### Ethical approval

All experimental procedures were done after approval by the ethics committee of Shahid Beheshti University of Medical Sciences (Approval ID: IR.SBMU.MSP.REC.1399.131).

## Results

### Cancer cell culture

Hela and MCF-7 cells were incubated in a culture medium containing RPMI, 10% FBS, and 100 U/ml penicillin/streptomycin. After three cell passages and 80% confluency, cancer cells viability and morphology were assessed by trypan blue and inverted microscope, respectively. The viability of cancer cells was more than 95%.

### Sensitivity of hela and MCF-7 cell lines to PTX

**Figure 1:**
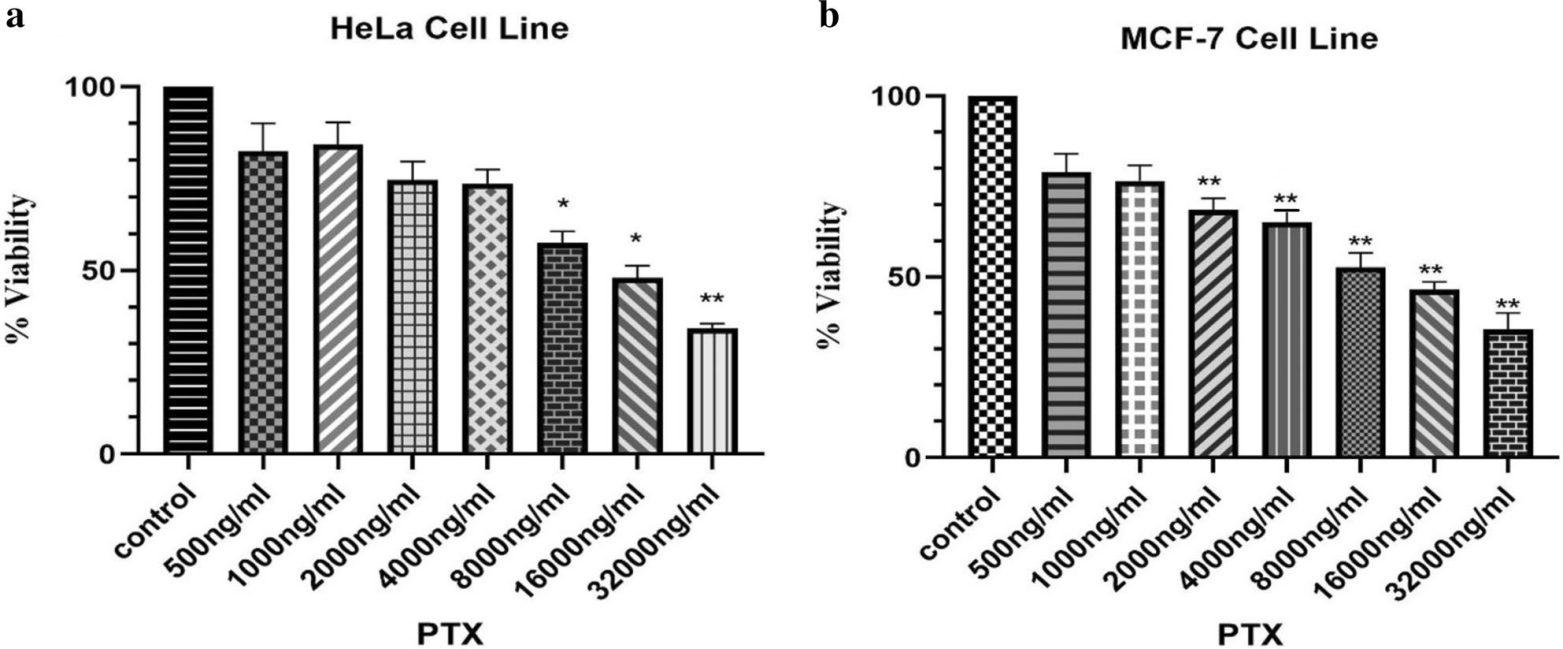
The sensitivity of HeLa and MCF-7 cell lines to PTX. (**a**) HeLa cells (10^4^ cells) showed reduced viability from the concentration of 8000 ng/ml of PTX. (**b**) MCF-7 cells (10^4^ cells) showed decreased cell viability from 2000 ng/ml of PTX. *P ≤ 0.05; **P ≤ 0.01.

To evaluate the sensitivity of HeLa and MCF-7 cancer cells to PTX, we used an MTT assay to assess cell viability after exposure to serial concentrations of PTX (500, 1000, 2000, 4000, 8000, 16000, 32000 ng/ml). The results showed that both Hela and MCF-7 are partially sensitive to PTX in a concentration-dependent manner. HeLa cells displayed cytotoxic effects of PTX from the concentration of 8000 ng/ml, while the viability of MCF-7 cells reduced from 2000 ng/ml of PTX (Fig. 1).

### Isolation and characterizing of hAECs from amniotic membrane

**Figure 2:**
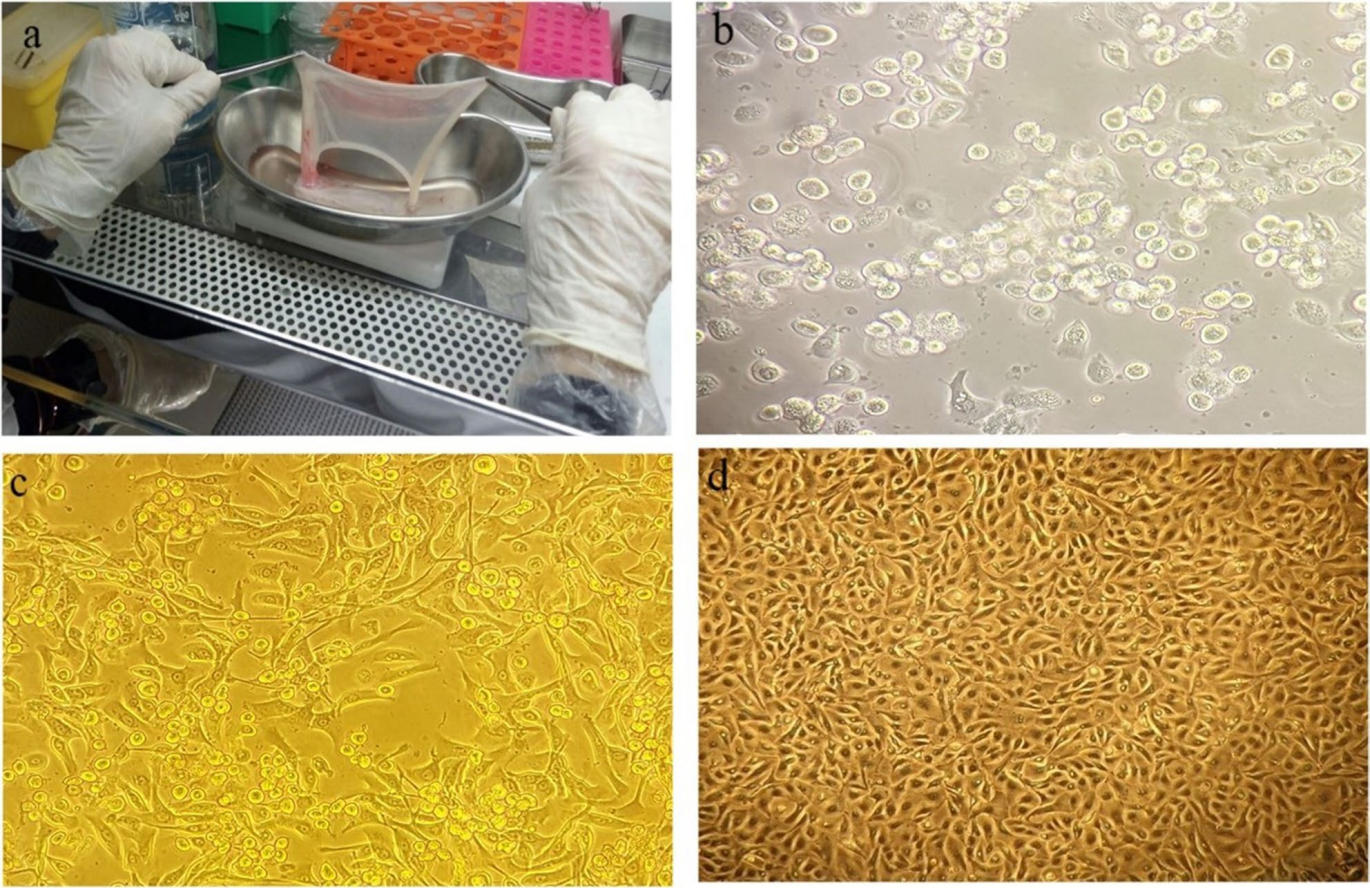
Isolation of hAECs. (**a**) An amniotic membrane is provided from the human placenta under sterile conditions. (**b**) hAECs immediately after isolation on day zero. (**c**) hAECs three days after culture under standard conditions. (**d**) hAECs ultimately reached confluency.

hAECs were isolated from amnion membranes of placentae belonging to healthy women's term gestational age pregnancies after double-step enzymatic digestions with trypsin. The viability and morphology of isolated cells were evaluated by trypan blue and inverted microscope, respectively. The cell viability assay showed that more than 95% of isolated cells were viable. On the day of isolation, hAECs mostly displayed round shapes; however, some polygonal hAECs were observed. The cells were elongated and spindle-shaped without granulation after three days of culture. Finally, cells reached complete confluency on the seventh day (Fig. 2).

**Figure 3:**
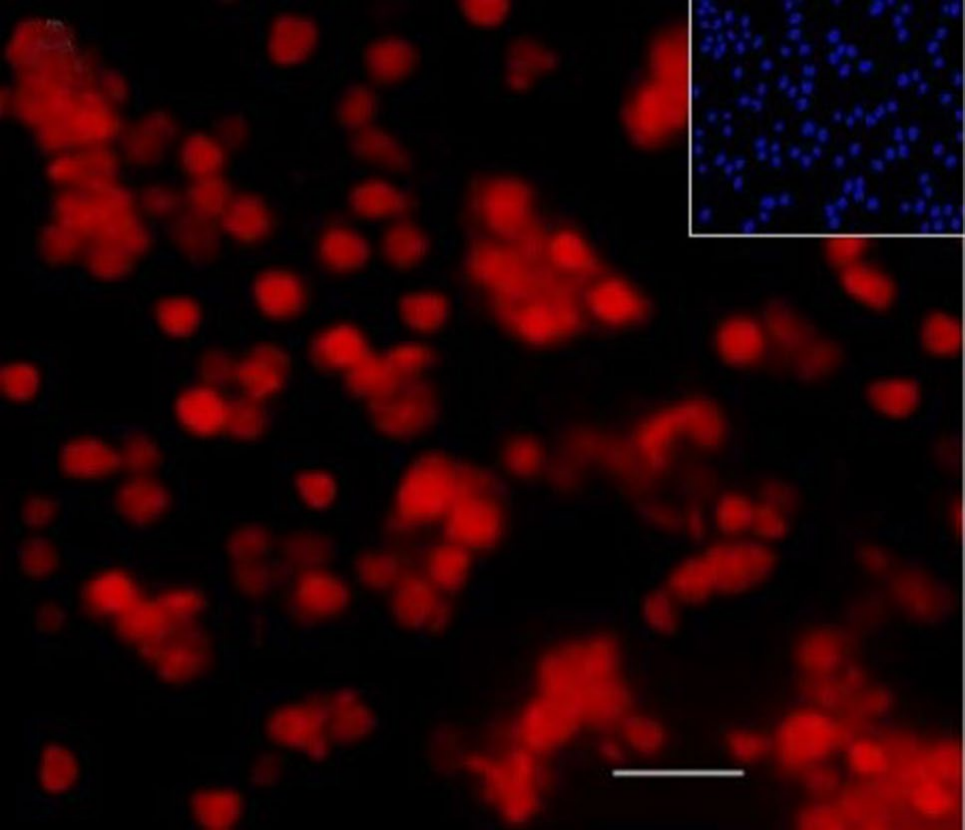
The delineation of newly isolated hAECs with the pan-cytokeratin marker. The inset figure (upper right) shows DAPI nuclear staining of the same field. Scale bar: 50 μm.

The characteristics and purity of hAECs were assessed by immunocytochemistry test showing pan-cytokeratin marker as hAECs consistently expressed pan-cytokeratin^26^. The test results indicated that more than 98% of the isolated cells were positive for the pan-cytokeratin marker (Fig. 3).

### hAECs were resistance to cytotoxic effects of PTX

**Figure 4:**
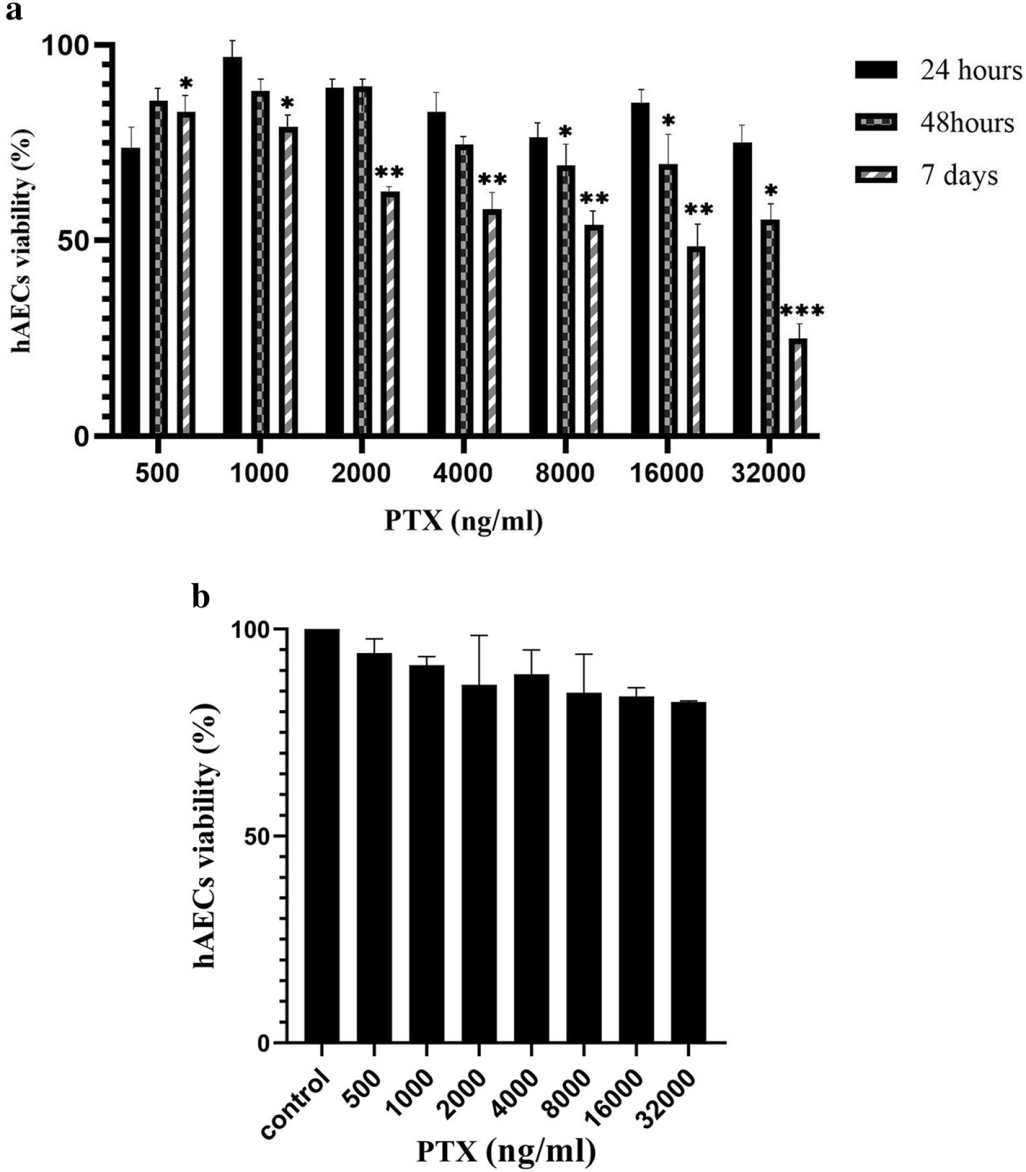
Effects of PTX on the viability of hAECs. (**a**) PTX treatment for 24 h did not change hAECs (10^4^ cells) viability even at the highest concentrations. After 48 h, hAECs (10^4^ cells) showed reduced viability only in 8000, 16,000, and 32,000 ng/ml (2000 cells). Anti-proliferation assay (seven days) showed reduced proliferation of hAECs after exposure to PTX in all concentrations (2000 cells). (comparison was made with control group of each time point) (**b**) Seven days drug-free period after 24 h of PTX exposure showed preserved proliferation ability of hAECs (donor number = 6). *P ≤ 0.05; **P ≤ 0.01; ***P ≤ 0.001.

In order to determine the appropriate period for PTX loading on hAECs, the viability of these cells was assessed after exposure to PTX for 24 hours, 48 hours, and 7 days. The results of the viability evaluation showed that hAECs were highly resistant to PTX cytotoxicity after 24 hours of exposure to PTX. The viability of PTX-treated hAECs did not significantly differ from control in all concentrations. Their viability remained near 80%, even at the highest (32000 ng/ml) assessed PTX concentration (Fig. 4a). On the other hand, after 48 hours of exposure to PTX, hAECs displayed lowered viability in concentrations of 8000, 16000, and 32000 ng/ml (Fig. 4a). The results of the 7 days treatment of the hAECs with PTX demonstrated that hAECs are sensitive to the anti-proliferative impacts of PTX according to the dose-dependent manner (Fig. 4a). Based on the viability data, 24 hours treatment of hAECs with PTX is the optimal duration due to the highest resistance of these cells in this period.

**Figure 5:**
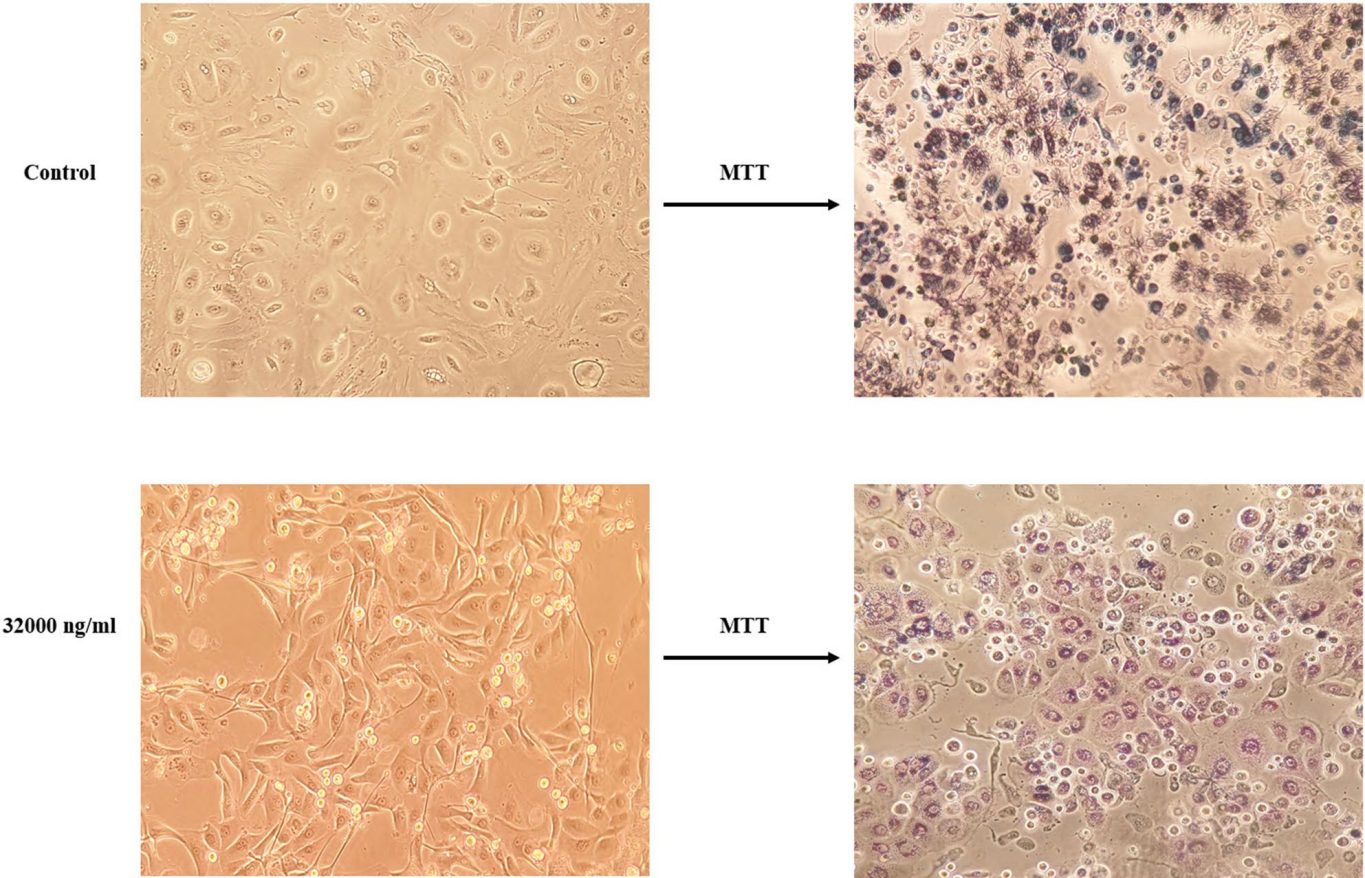
the effects of PTX treatments on hAECs morphological characteristics. After 24 h of treatment with the highest tested concentration of PTX, hAECs showed shrinkage and some granulations. However, there were no significant signs of cell death (left column). Utilizing 3-(4,5-dimethylthiazol-2-yl)-2,5-diphenyltetrazolium bromide (MTT) for cell visualization with insoluble formazan crystals showed that the mitochondria of both PTX treated and control cells converted MTT to formazan (right column).

In order to assess the proliferative potential of hAECs following 24 hours loading period, the growth ability of hAECs was evaluated after seven days of the loading process. In this regard, hAECs displayed normal proliferation capacity seven days after PTX removal from the culture environment. These cells preserved their proliferation ability even at the highest tested concentration (32000 ng/ml), which provides appropriate proliferative capacity for tissue regeneration. (Fig. 4b). Besides, the morphological alterations of hAECs in the highest tested concentration (32000 ng/m) after 24 hours with the signs of insoluble formazan formation are shown in Figure 5.

### Effect of P-gp inhibitor on PTX sensitivity and uptake/release ability of hAECs

**Figure 6:**
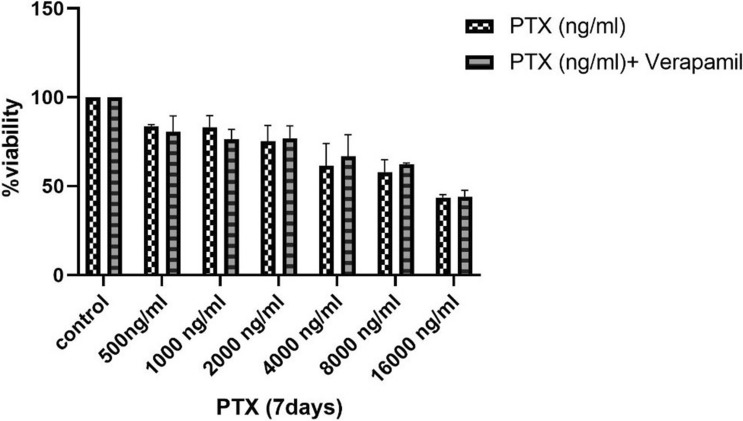
Resistance mechanism of hAECs against PTX. Co-treatment of hAECs (2000 cells) with PTX and verapamil, a *P*-gp inhibitor, showed that verapamil did not reduce the viability of hAECs after exposure to PTX. *P ≤ 0.05; **P ≤ 0.01; ***P ≤ 0.001.

Some studies have proposed *P*-gp as an underlying drug efflux pump in cells that can induce chemotherapeutic drug resistance^27,28^. In order to investigate whether *P*-gp is responsible for hAECs PTX resistance by outpouring the drug, we blocked *P*-gp by verapamil as a *P*-gp pump inhibitor. Then, we evaluated whether blocking *P*-gp could alter hAECs sensitivity to PTX. The results showed that verapamil did not affect hAECs sensitivity to the anti-proliferative activity of PTX (Fig. 6).

### hAECs Incorporated and Released PTX

**Figure 7:**
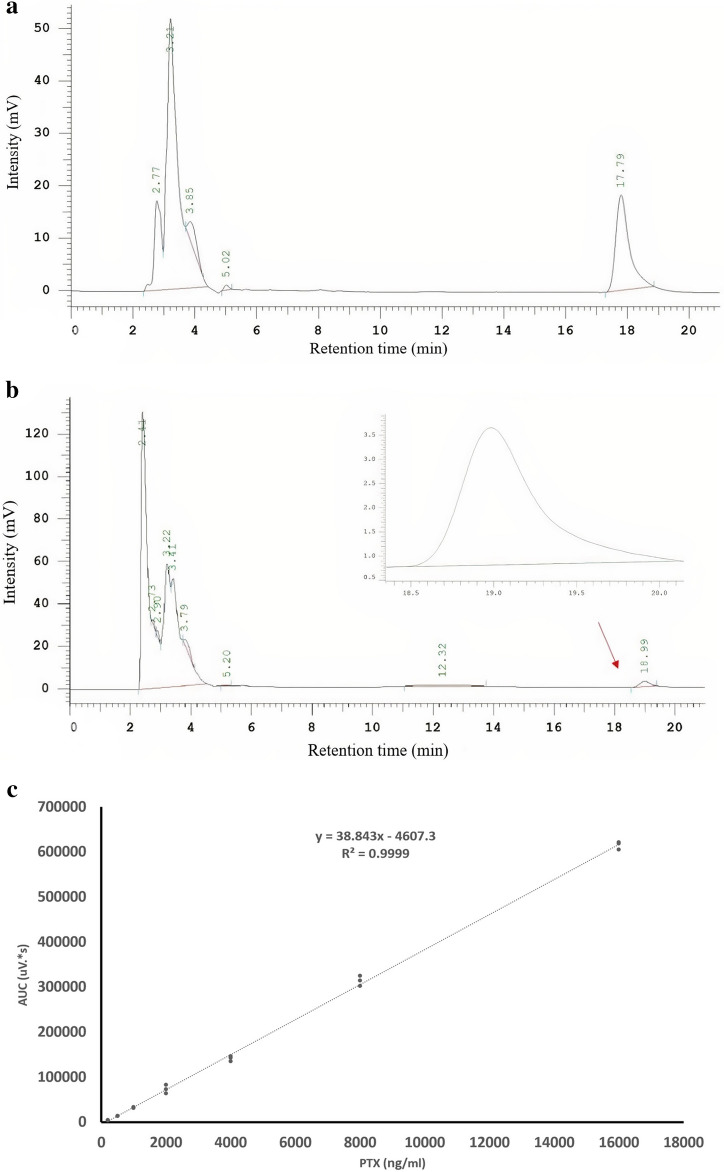
HPLC curve of PTX. (**a**) The PTX-related peak was observed near 18 min in the control culture medium. (**b**) the peak of PTX appeared at 18.99 min in the hAEC-PTX/CM sample. (**c**) The standard calibration curve of pure PTX which shows y = 38.843x—4607.3, R^2^ = 0.9999.

After observing the resistance of hAECs against cytotoxic effects of PTX after 24 hours of treatment, we then pursued to investigate whether they could incorporate and release the drug in the environment and which concentration of PTX is appropriate for the loading process. Considering the inefficiency of 500 and 1000 ng/ml of PTX to reduce the viability of evaluated cancer cell lines and also the high toxicity of 32000 ng/ml concentration for hAECs, the ability of hAECs to incorporate and release PTX was evaluated by priming the cells with 2000, 4000, 8000 and16000 ng/mL of PTX. We tested the conditioned medium of primed cells (hAEC-PTX/CM) after 24, 48, 72, and 96 h of their subculture. The pure PTX allowed us to define a standard dose-response curve on which the amount of PTX in both cell lysate and CM was estimated. Standard HPLC chromatograms showed that the drug was eluted with a peak at 18 min. An identical peak retention time for PTX was eluted by processing both lysate and CM from hAECs loaded with PTX (Fig. 7a,b). A calibration curve (200, 500, 1000, 2000, 4000, 8000, 16000 ng/ml) was prepared that was used to quantify paclitaxel (y = 38.843x–4607.3, R^2^ = 0.9999) (Fig. 7c). Based on the standard deviation of the Y-intercepts and formulae 1 and 2, LOD was calculated as 110.87 ng/ml, while LOQ was 332.6 ng/ml. HPLC analysis revealed the presence of other nonspecific peaks due to compounds produced by cells; however, they do not interfere with the presence of PTX. These peaks are also present in the chromatogram of control lysate and medium.

**Figure 8:**
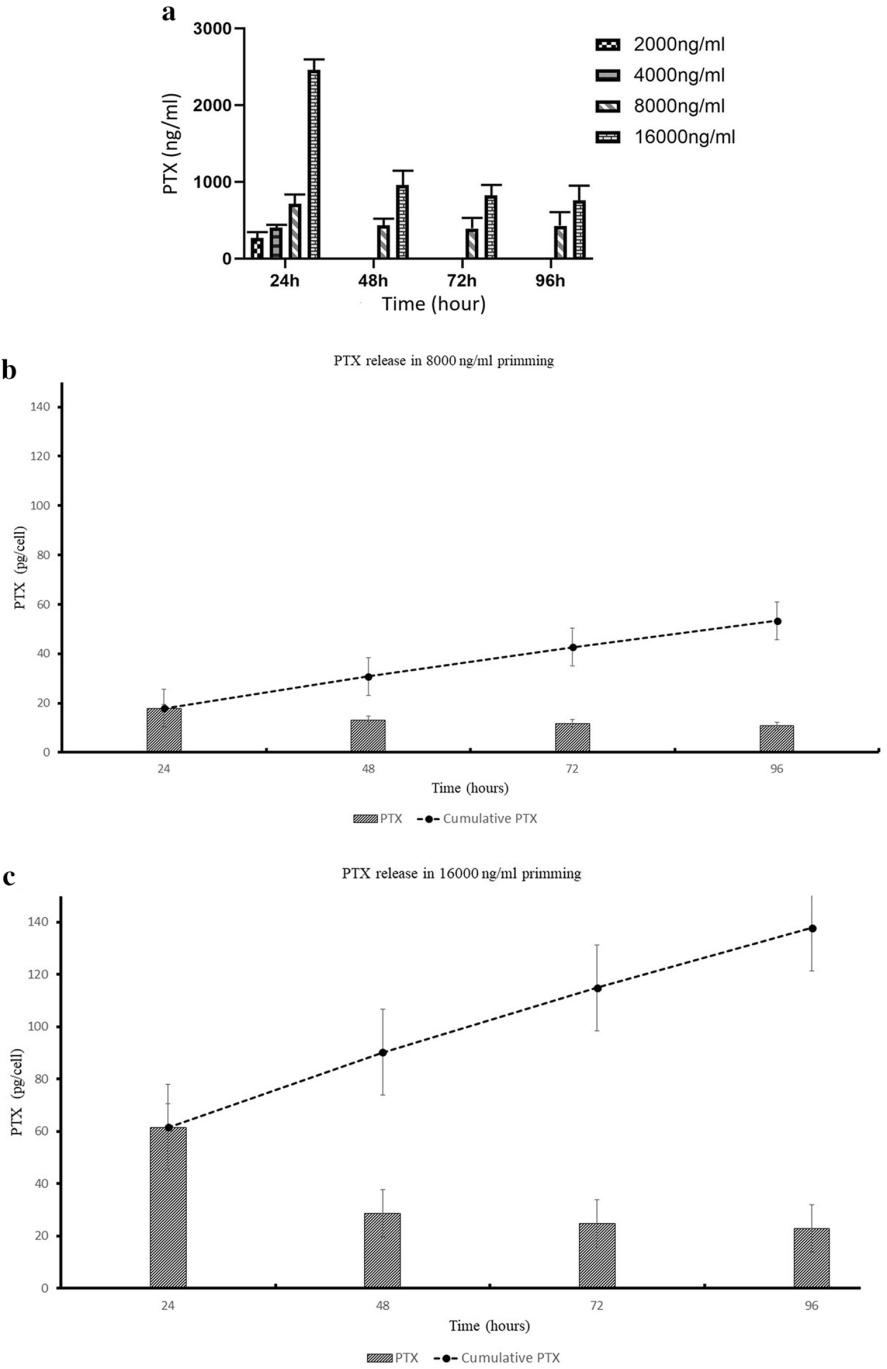
PTX release by hAECs. (**a**) Evaluating the amounts of PTX in CM of PTX-loaded hAECs showed that these cells released PTX in their CM for up to 96 h. Although hAECs released PTX during the first 24 h in all treated concentrations, only 8000 and 16,000 ng/ml-treated hAECs released PTX up to 96 h. (**b**, **c**) Evaluating the release pattern of hAECs loaded with PTX at 8000 ng/ml (**a**) and 16,000 ng/ml (**b**), hAECs released PTX for 96 h in a steady pattern which was more prominent in the first 24 h. The dotted line shows cumulative amounts of released PTX from 24 to 96 h, showing a steady and sustained release pattern for PTX.

In HPLC evaluation of hAEC-PTX/CM in serial collection times, PTX was not detected in hAEC-PTX/CM primed with 2000 ng/ml and 4000 ng/ml after the first 24 hours. The release of PTX was highest after 24 hours in all concentrations; however, hAECs released PTX in a steady pattern up to 96 hours (Fig. 8). The uptake efficacy (PTX uptake proportion) of primed hAECs differed between concentrations. The heights PTX uptake rate occurred in 8000 ng/ml treatment, in which 14.23% of available PTX was up-taken, while the least was for 2000 ng/ml treatment, which was 3.38%. HPLC analysis on lysate of primed hAEC-PTX after 96 hours showed that a proportion of the internalized PTX was not released into the environment and remained inside primed hAECs. We could not detect PTX in hAEC-PTX/LYS, which was loaded with 2000 ng/ml of PTX, which can be due to the low amount of PTX. However, hAECs loaded with 4000, 8000, and 16000 ng/ml of PTX could release the most of internalized PTX (88% for 4000 ng/ml loaded and 87% for 8000 and 16000 ng/ml). Considering the highest uptake and release efficacy of hAECs loaded with ng/ml of PTX and also a complete block of hAECs proliferation, but not significant cell toxicity, this concentration was determined as the optimum loading dose.

### Primed hAECs reduced the proliferation of cancer cells

**Figure 9:**
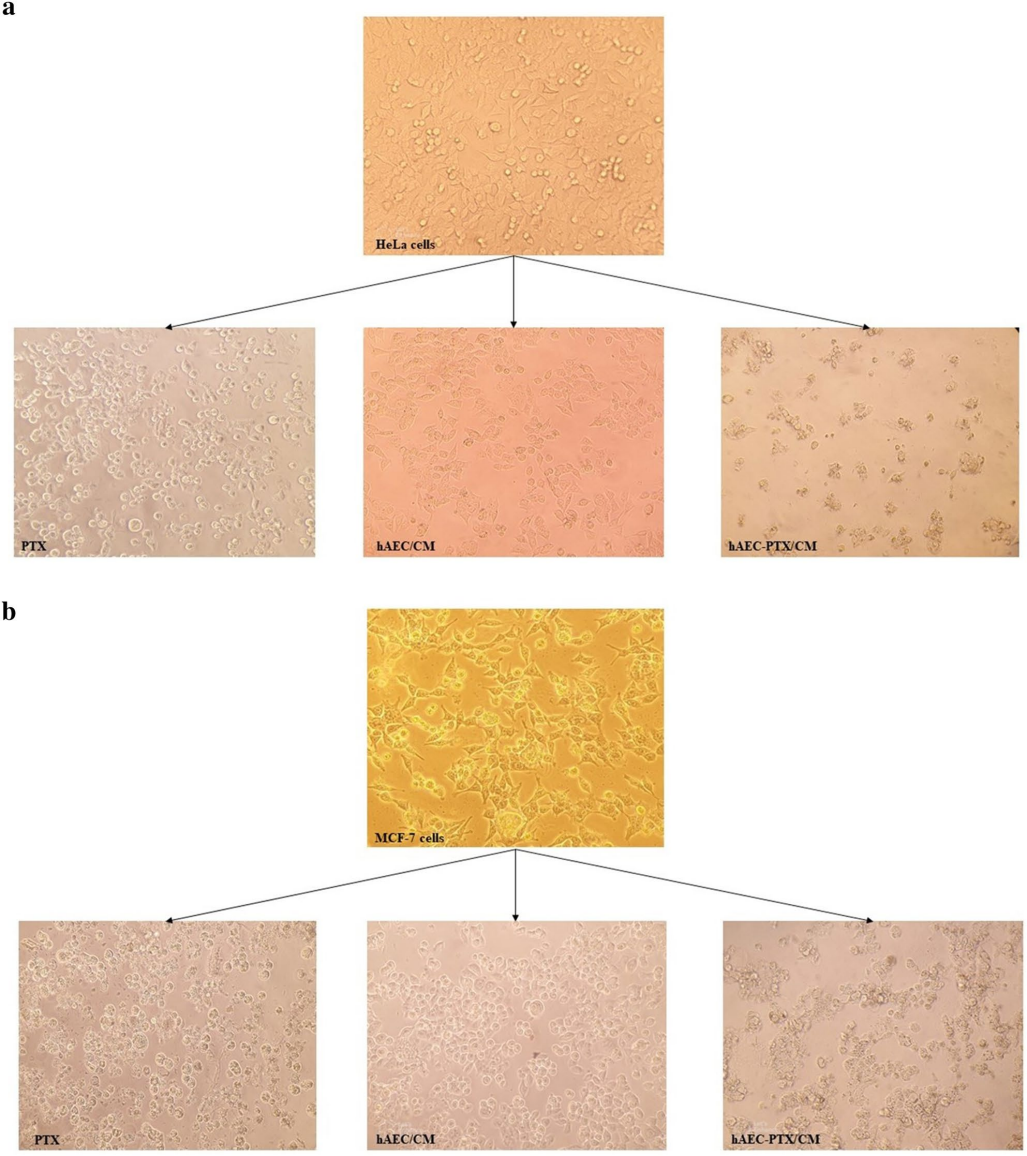
The morphological changes of MCF-7 and HeLa cancer cells after exposure to pure PTX, hAEC/CM, and hAEC-PTX/CM. (**a**, **b**) MCF-7 and HeLa cancer cells turned granulated, shrunk, and round shape after treatment with pure PTX. Treatment with hAEC/CM resulted in roundish change, but less granulation was observed. The cancer cells which were treated with hAEC-PTX/CM displayed high granulation, reduced attached cells, and round shape.

In order to explore whether the PTX containing secretome released from hAECs was adequate to inhibit tumor cell proliferation, hAEC-PTX/CM of all concentrations were collected 24 hours after priming. The anti-proliferative ability of hAECs-CMs and hAEC-PTX/CM was evaluated on partially sensitive cancer cell lines, HeLa and MCF-7, that we evaluated their sensitivity to pure PTX with IC50: 2123 ng/ml for HeLa and IC50: 2622 ng/ml for MCF-7 cells. The hAEC-PTX/CMs of all concentrations produced a dose-dependent anti-proliferative effect on HeLa and MCF-7 cancer cell lines. Considering the highest uptake and release efficacy in 8000 ng/ml loading concentration, the results of hAEC-PTX/CM in this concentration are shown in Table1. The CM of 8000 ng/ml primed hAECs produced a strong dose-dependent anti-proliferative effect on MCF-7 (IV50: 144.6 μL) and HeLa (IV50: 98.37 μL). The range of anti-proliferative effects of hAEC-PTX/CM released from 10^5^ primed hAECs was equivalent to those attained with pure PTX at doses from 114.35 to 2882.05 ng/ml for MCF-7 cancer cells and from 99.18 to 2994.26 ng/ml for HeLa cancer cells. Considering the calculated concentrations of PTX in hAEC-PTX/CM, the IV50 of hAEC-PTX/CM on MCF-7 and HeLa cells contained 644 ng/ml and 438 ng/ml PTX, respectively. Compared to IC_50_ of pure PTX on these cell lines, hAEC-PTX/CM produced 3.3 and 4.8 times stronger anti-proliferative effects on MCF-7 and HeLa, respectively. The morphological changes of HeLa and MCF-7 cells after exposure to pure PTX, hAEC/CM, and hAEC-PTX/CM are shown in Figure 9.

**Table 1:** Anti-proliferative effects of hAEC-PTX/CM on HeLa and MCF-7 cell line.

	HeLa cell line		MCF-7 cell line	
	hAEC-PTX/CM	Pure PTX	hAEC-PTX/CM	Pure PTX
IC50 (ng/ml)	–	2123	–	2622
IV50 (μL)	98.37	–	144.6	–
IV50 PTX content (ng/ml) *	438	–	644	–

*The concentration of PTX present in the IV50 dilution of hAEC-PTX/CM.

**Figure 10:**
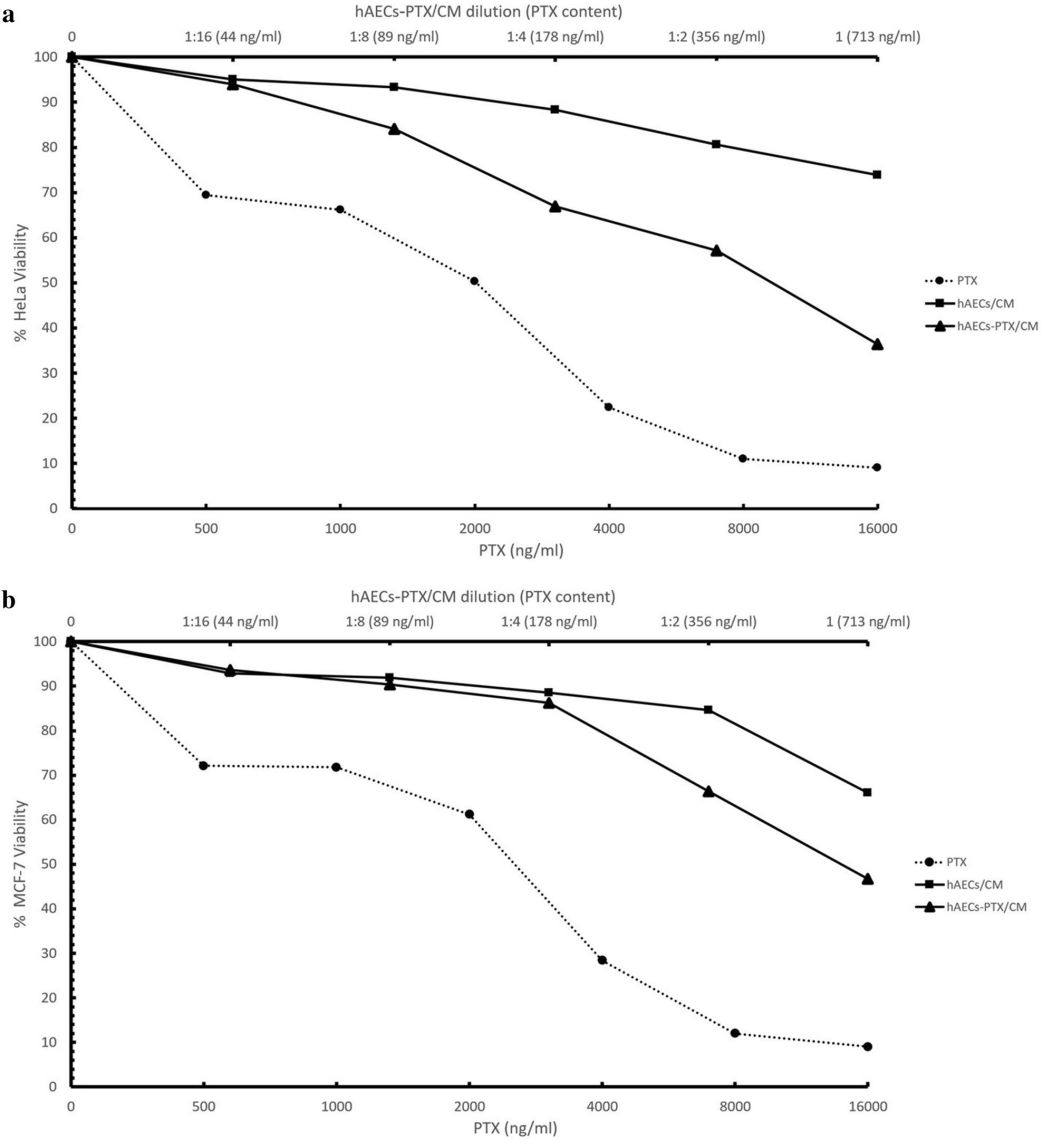
The anti-proliferative effects of hAECs-CM and hAEC-PTX/CM on MCF-7 and HeLa cancer cells. (**a**) Serial dilutions of pure PTX, hAECs/CM and hAEC-PTX/CM (collected from 10^5^ hAEC-PTX) induced anti-proliferative effect on HeLa cancer cells. The amounts of containing PTX in each dilution are shown in parenthesis (above the hAECs-PTX/CM dilution axis) (**b**) Serial dilutions of pure PTX, hAECs-CM, and hAEC-PTX/CM (collected from 10^5^ hAEC-PTX) induced anti-proliferative effect on MCF-7. The below axis and dotted line are related to the amounts of pure PTX.

In order to evaluate the part of innate anti-neoplastic effects of hAECs, the anti-proliferative effects of hAECs-CM were evaluated on MCF-7 and HeLa cancer cell lines. hAECs-CMs displayed anti-proliferative effect on MCF-7 and HeLa equivalent to pure PTX doses from 322.62 to 1160.58 ng/ml and from 81.69 to 313.26 ng/ml, respectively (Fig. 10).

### Primed hAECs enhanced apoptosis process

**Figure 11:**
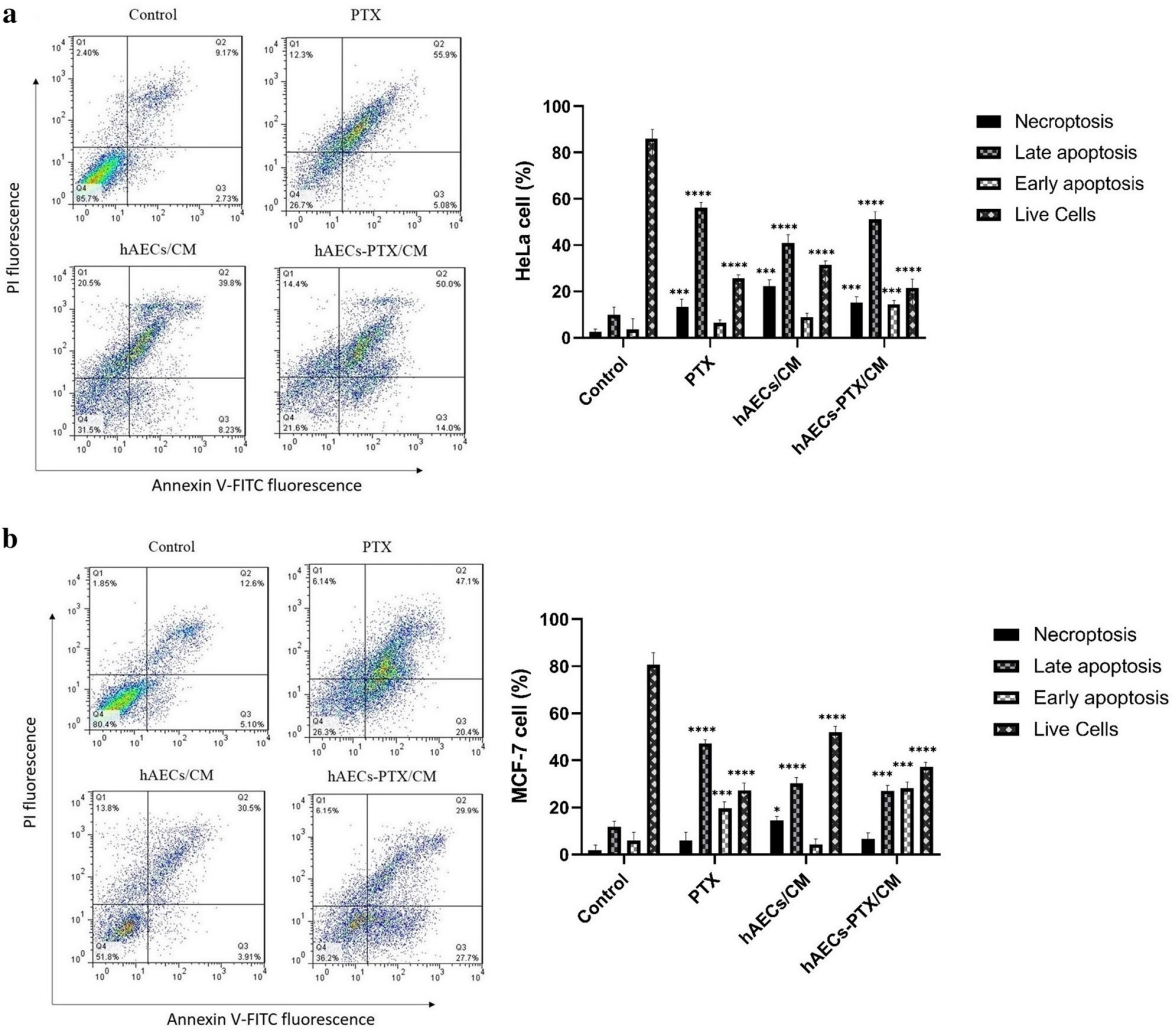
The effects of primed hAECs on the apoptosis process of HeLa and MCF-7 cancer cells. Annexin V-FITC and PI staining were used to evaluate apoptosis in pure PTX, hAECs/CM, and hAEC-PTX/CM treated HeLa (**a**) and MCF-7 (**b**) cells. Besides, untreated HeLa and MCF-7 cells were considered as the control groups (n = 5). Considering the qualitative apoptosis test, the upper left quadrant (Q1) indicates Annexin V^negative^ PI^positive^ cells related to the necrosis process, the upper right quadrant (Q2) shows Annexin V^positive^ PI^positive^ related to late apoptosis, the lower right quadrant (Q3) displays Annexin V^positive^ PI^negative^ related to early apoptosis, and finally left lower quadrant shows Annexin V^negative^ PI^negative^ associated with viable cells. The percentages of viable, early apoptotic, late apoptotic, and necrotic cells are shown in the right charts. Statistic significance was obtained from comparison among experimental groups and control group. *P ≤ 0.05; **P ≤ 0.01; ***P ≤ 0.001; ****P ≤ 0.0001.

In order to reach better insight into the anti-proliferative related mechanism of hAEC/CM and hAEC-PTX/CM, we carried on the apoptosis analysis. After treatment of HeLa and MCF-7 cancer cells with pure PTX, hAEC/CM, and hAEC-PTX/CM, the number of Annexin V-stained cells was determined utilizing flow cytometry. Regarding the results, apoptotic cells were significantly higher in the hAEC-PTX/CM group compared with the control group in the HeLa (64% vs 11.9) and MCF-7 (67.5% vs 17.7) groups. Besides, hAECs-PTX/CM significantly reduced the viability of cancer cells from 85.7 to 21.6 and 80.4 to 26.3 in HeLa and MCF-7 cells, respectively (Fig. 11).

## Discussion

Considering the innate anti-neoplastic effects of hAECs, we evaluated the possibility and appropriate condition of loading paclitaxel on these cells. Besides, the possibility of using hAECs as a drug delivery system to the tumor and the anti-proliferative effects of their secretome were evaluated on the partially sensitive cancer cell lines, MCF-7 and HeLa.

We showed that hAECs are highly resistant to cytotoxic effects of PTX during 24 hours of treatment. Previous studies have demonstrated the resistance of some types of stem cells, including MSCs to PTX. For instance, it has been shown that human bone marrow-derived MSCs displayed high resistance against cytotoxic effects of PTX up to 10,000 ng/mL^29,30^. Another study showed dental pulp stem cells presented higher paclitaxel resistance compared to human bone marrow-derived MSCs^31^. In order to evaluate the resistance of placenta-derived stem cells, Bonomi et al. showed that human placenta-derived amniotic membrane MSCs could tolerate cytotoxic effects of PTX even at concentrations up to 10,000 ng/mL. However, we demonstrated that hAECs remained viable during exposure to PTX even at the highest concentration of 32000 ng/ml. In order to find an appropriate primming time, we tested the effects of 48 hours and seven days of treatment with PTX on the viability of hAECs. We demonstrated that the viability of hAECs decreased after 48 hours in concentrations of 8000, 16000, and 32000 ng/ml. Besides, hAECs were sensitive to anti-proliferative effects of PTX in all concentrations after seven days of treatment. Almost all previous studies have confirmed that extended exposure to PTX significantly reduces the viability of other stem cell types in a dose-dependent manner^32,33^. Considering the importance of proper biological activity for drug loading and releasing, 24 hours period for the loading process was selected from previous studies^24,34^.

In order to investigate the mechanism of hAECs resistance to PTX, some studies have proposed *P*-gp efflux pumps as a possible resistance mechanism^24,34^. It has been shown that the *P*-gp efflux pump, as a member of ATP-binding cassette transporters (ABC transporters), increases the resistance to chemotherapeutic drugs in various normal or neoplastic cells^27,35^. Herein, we have shown that *P*-gp efflux pumps did not participate in the chemoresistance features of hAECs. These results are along with the previous studies that revealed inhibiting *P*-gp by verapamil could not increase the sensitivity of MSCs to PTX^30,34^. Proposing other mechanisms of PTX resistance such as HSPs, alteration of tubulin isotypes, and behavior of proteins participating in apoptosis could help to understand better the underlying mechanism of hAECs chemoresistance in future studies^8^.

Besides the PTX resistance of hAECs in 24 hours treatment, whether hAECs preserve their innate proliferative potential is another critical question that should be evaluated after PTX loading of hAECs. It has been indicated that a favorable regeneration process results from appropriate cell proliferation and cellular differentiation to restructure injured tissues^36^. In this study, we have shown that a 24 hours period of PTX loading could not affect the proliferation ability of hAECs. Thus, hAECs would participate in chemotherapy-induced tissue damage by regenerating damaged tissues.

PTX uptake and release ability is the mainstay of probable using hAECs as a drug delivery system. This study showed that hAECs could uptake and release PTX when loaded with 2000, 4000, 8000, and 16000 ng/ml concentrations. Afterward, defining an appropriate loading concentration is pivotal to prime hAECs with PTX. In this regard, the PTX uptake and release efficacy of hAECs were assessed by loading with different concentrations and also collecting the hAEC-PTX/CM during other time points. The result revealed that hAECs could uptake PTX in 8000 ng/ml with the highest efficacy, in which 14.23% of available PTX was up-taken. Previous studies have shown that other stem cells, especially MSCs from different sources, can be loaded with PTX. For instance, Pessina et al. showed that human bone marrow-derived MSCs can uptake about 8% of the total drug in the culture medium of PTX, which was approximately 2.7 pg/per cell^34^. In another study, human olfactory bulb neural stem cells primed with PTX incorporated 0.19 pg of the drug per cell^25^. Besides, PTX uptake was reported in some stem cells derived from different sources such as adipose tissue and gingiva^37,38^. However, an in vivo study on orthotopic glioblastoma xenografts showed that PTX loading in MSCs derived from adipose tissues did not considerably improve the antitumor effects of these cells^37^. However, our in vitro study showed that loading PTX on hAECs has enhanced their anti-cancer potency. Conclusively, it seems that the amount of incorporated PTX and the efficacy of administring PTX-loaded cells depends on the type and source of utilized stem cells. Besides, further studies are essential to evaluate the effects of drug loading on the anti-neoplastic outcomes of hAECs.

A sustain releasing PTX delivery system provides an efficient concentration for suppressing tumor cell growth and lessens the systemic collateral toxicity of this drug. Our HPLC analysis showed that hAECs could release about 87% of PTX in a steady-state manner during 96 hours. It is notable that during the first 24 hours, hAEC-PTX released approximately 47% of incorporated PTX and continued to release PTX for 96 hours. Consistent with our results, prior studies revealed that MSCs loaded with PTX released the drug in a time-dependent manner. It has been shown that during the first 24 hours, MSCs derived from human adipose tissue and bone marrow secreted the majority of incorporated PTX, and negligible amounts of PTX were detected for the next 48 and 144 hours. The results showed that MSCs from bone marrow are more potent than adipose tissue to uptake and release higher amounts of PTX^39^. Another study showed that almost 52% of incorporated PTX was released from human olfactory bulb neural stem cells during the first 24 hours after loading with PTX^40^. Considering the lipophilic nature of PTX, simple diffusion could be proposed as a possible internalization mechanism of PTX by hAECs^30^. Also, some studies suggested that PTX is released through extracellular vehicles (EVs), especially exosomes, that play essential roles both as the biological carriers for drugs and endogenous particles^29^. It seems that the source of stem cells and drug type influence the drug uptake and release capacity. Therefore, further studies can shed light on the possible PTX incorporation and release mechanism by hAECs.

In order to investigate whether the PTX released from hAEC-PTX was enough to reduce tumor cell proliferation, we tested the anti-proliferative effects of hAEC-PTX/CM on HeLa and MCF-7 cancer cell lines which are partially sensitive to PTX. Our results showed that hAEC-PTX/CM of 10^5^ primed hAECs induce strong anti-proliferative effects on MCF-7 and HeLa cancer cells. The anti-proliferative effects of hAEC-PTX/CM on MCF-7 and HeLa cancer cells were equal to 114.35 to 2882.05 ng/ml of pure PTX for MCF-7 cells and from 99.18 to 2994.26 ng/ml of pure PTX for HeLa cells. These results were along with previous studies in which CM of PTX-primed MSCs induced potent growth inhibition on PTX-sensitive human prostate cancer and glioblastoma cells equal to 25 ng/mL of pure PTX^34^. Another study showed that CM of paclitaxel-loaded amniotic membrane-derived MSCs could produce a dose-dependent anti-proliferation effect on very sensitive CFPAC-1 cells, a ductal pancreatic adenocarcinoma cell line^24^. In our study, the maximum anti-proliferative effect of hAEC-PTX/CM was 55% cancer cell death for both HeLa and MCF-7. Considering the limited number of hAECs loaded with paclitaxel (10^5^ cells), more anti-proliferative outcomes can be expected by administering higher numbers of hAEC-PTX. In this regard, many clinical trials administered high numbers of hAECs (from one to a hundred million cells) in different pathological situations^41^.

In order to evaluate the part of innate anti-neoplastic effects of hAECs, the anti-proliferative effects of hAECs-CM and hAEC-PTX/CM were compared. The results showed that both hAECs-CM and hAEC-PTX/CM could induce anti-proliferative effects on MCF-7 and HeLa cells; however, loading of PTX could enhance the anti-proliferative effects of hAECs. Along with our study, it has been shown that hAECs-CM significantly induced apoptosis and necrosis in MCF-7 cells^42^. However, we have demonstrated that loading PTX on hAECs can synergistically increase their anti-proliferative effects.

## Conclusion

In conclusion, our data indicated that hAECs are resistant to cytotoxic effects of PTX, which was not mediated by the efflux ability of *P*-gp pumps. The HPLC analysis showed that the optimum method for loading PTX on hAECs is 24 hours of exposure to 8000 ng/ml of PTX. It is also indicated that the loading PTX did not affect the proliferation ability of hAECs. The condition media of PTX induce anti-proliferative effects on MCF-7 and HeLa cell lines. Besides, loading PTX on hAECs can synergistically increase these cells' anti-neoplastic efficacy. Therefore, using PTX-loaded hAECs can increase the effectiveness of cell-mediated cancer treatment compared to previously used cells, including MSCs.

## Data Availability

This article contains no supplementary information. The data that support the findings of this study are included in this article; further inquiries can be directed to the corresponding author.
